# Factors influencing IUCN threat levels to orchids across Europe on the basis of national red lists

**DOI:** 10.1002/ece3.2363

**Published:** 2016-08-04

**Authors:** Tiiu Kull, Ulvi Selgis, Miguel Villoslada Peciña, Mirjam Metsare, Aigi Ilves, Kadri Tali, Kalev Sepp, Kalevi Kull, Richard P. Shefferson

**Affiliations:** ^1^ Institute of Agricultural and Environmental SciencesEstonian University of Life SciencesKreutzwaldi 5Tartu51014Estonia; ^2^ Institute of Philosophy and SemioticsUniversity of TartuJakobi 2Tartu50090Estonia; ^3^ Department of General Systems StudiesUniversity of Tokyo3‐8‐1 KomabaMeguro‐kuTokyo153‐8902Japan

**Keywords:** Endangered, European orchids, IUCN national red lists, land cover, nectarless, rhizomatous

## Abstract

The red list has become a ubiquitous tool in the conservation of species. We analyzed contemporary trends in the threat levels of European orchids, in total 166 species characterized in 27 national red lists, in relation to their reproductive biology and growth form, distribution area, and land cover where they occur. We found that species in central Europe are more threatened than those in the northern, southern, or Atlantic parts of Europe, while species were least threatened in southern Europe. Nectarless and tuberous species are significantly more threatened than nectariferous and rhizomatous taxa. Land cover (ratios of artificial land cover, area of pastures and grasslands, forests and inland wetlands) also significantly impacted the threat level. A bigger share of artificial land cover increases threat, and a bigger share of pasture and grassland lowers it. Unexpectedly, a bigger share of inland wetland area in a country increased threat level, which we believe may be due to the threatened nature of wetlands themselves relative to other natural land cover types. Finally, species occurring in multiple countries are on average less threatened. We believe that large‐scale analysis of current IUCN national red lists as based on their specific categories and criteria may particularly inform the development of coordinated regional or larger‐scale management strategies. In this case, we advocate for a coordinated EU protection and restoration strategy particularly aimed at central European orchids and those occurring in wetland area.

## Introduction

The orchid family (Orchidaceae) is among the most diverse within the angiosperms (Mabberley 1997). However, this diversity is matched with rarity, in that most orchid species are threatened or endangered in the wild (Cribb et al. 2003). The relative rarity of members of this family has resulted in it being given special conservation status and protection in many countries (Bilz et al. 2011). Nonetheless, this protection has not staved off a general decline in the orchid flora of Europe (Jacquemyn et al. 2005; Kull and Hutchings 2006) and even East Asia (Kobori and Primack 2003; Yang et al. 2004).

The rarity of orchids is most certainly exacerbated by the interests of collectors and “orchid hunters.” However, it is clear that the biology of orchids contributes strongly to their rarity. Fruit set is often low in orchids, with nectarless species fruiting significantly less than nectariferous species worldwide (Neiland and Wilcock 1998). Natural fruit set in some *Cypripedium* species, for example, is typically lower than 10% (Primack and Stacy 1998; Shefferson et al. 2014). Orchid seeds are dust seeds that generally require symbiotic contact with an appropriate mycorrhizal fungus, and germination rates in the wild are surprisingly low compared to many other plants (Rasmussen 1995). Intriguingly, short‐lived species have seemed to decline more precipitously than long‐lived species in Europe (Kull and Hutchings 2006), and of course, habitat loss and eutrophication have contributed strongly to increasing rarity. Because longevity may be related to life form (e.g., tuberous, rhizomatous), the overall below‐ground biology of orchid species could potentially predict conservation‐related fate (Kull 2002).

Europe has faced a strong human impact with the spread of settled agriculture over several millennia and in recent centuries rapid industrial development, resulting in a highly fragmented landscape (Bilz et al. 2011). The current land cover composition and patterns in Europe are partially the result of the many land transitions over recent decades, mainly driven by changes in farming, management systems, and demographic trends (Henle et al. 2008). Fuchs et al. (2012) identified major political decisions connected to significant land use changes, namely the postwar urbanization of Europe, the European timber shortage after World War II and European afforestation actions, cropland changes before and after the introduction of the Common Agricultural Policy and the fall of the Iron Curtain. Changes in world trade, the enlargement of the European Union, and the establishment of large‐scale ecological networks such as Natura 2000 have also played a very important role in European land use change dynamics in the past decade (Verburg et al. 2006).

Habitat destruction is found to be a major threat to orchids (IUCN/SSC Orchid Specialist Group, 1996). The majority of orchid species in Europe are connected to open landscape, especially grasslands and pastures, but also mires (Delforge 1995). Such landscapes have typically drawn large levels of human settlement and development (Finck et al. 2002). Urbanization, changing land use, and the intensification of agriculture are all particularly important impact factors (Stewart 1992; Tsiftsis et al. 2011).

The 21st century has seen the introduction of a new level in the half‐a‐century‐old red listing movement by applying more objective criteria (IUCN, 2001) for estimating species extinction risk (Rodrigues et al. 2006). National red lists provide fundamental information on trends in biodiversity loss (Hoffmann et al. 2008; Zamin et al. 2010). Red listing is very data sensitive (Robbirt et al. 2006), but fortunately through the activities of both professionals and amateurs orchids are well recorded in Europe (Kull et al. 2006). Recently, Maes et al. (2015) showed that red list assessments might differ among countries, particularly because these have not always been based on the latest IUCN red list categories and criteria (Sharrock and Jones 2009). The latest IUCN criteria for species assessment specify the quantitative rules based on populations’ sizes and ranges’ decline over 10 years or three generations (IUCN, 2001). However, even without going into details related to trends and range size among single species, some general patterns at the landscape level may be possible to be drawn with national red list data (Zamin et al. 2010). In marine environment, Dulvy et al. (2014) have successfully applied IUCN red list data on the analysis of extinction risks of sharks and rays in the global scale. In terrestrial ecosystems, especially for nonmigrating organisms like plants, national and regional red lists provide important additional information because these estimate the situation independently for different territories (Gärdenfors 2001).

The major goal of the current study was to analyze orchid species decline throughout Europe on the basis of recently published red lists, and to attempt to explain threat level with biological traits and landscape data. We also aimed to assess the effect of land cover on the threat level of orchid species in terms of the area extent of land cover types. From a basic approach, landscapes can be described by the composition and the distribution of patches (Guerry and Hunter 2002), and the specific composition of land cover and abundance of certain land cover types is related to the abundance and richness of both plant and animal species (Köllner 2000; Atauri and de Lucio 2001; Houlahan and Finlay 2003; Houlahan et al. 2006). Specific questions we asked include the following: (1) “What are orchid species’ threat‐level distributions (as characterizing the species dynamics trends) throughout the European region on the basis of national red lists data?” (2) “Which species and species groups are the most and least threatened?” (3) “Which biological traits are related to threat level?” (4) “What regional differences exist in threat level?” (5) “Are threat levels connected to the pattern of land cover types among countries?” (6) Finally, from these patterns, “can we draw any implications for conservation policies?”

We expected that threat level in older evolutionary groups is higher, species inhabiting many countries are less threatened within countries as well, and rhizomatous species are less threatened than tuberous species as shown earlier in much smaller‐scale studies (Kull and Hutchings 2006). Furthermore, as complicated pollination systems play an important role in orchid reproduction, we expected that there should be difference in threat level of nectariferous and nectarless species although it was not revealed by earlier more spatially restricted studies (Jacquemyn et al. 2005; Kull and Hutchings 2006). In northern countries, orchids reach their distribution range limit and therefore could be more threatened than in other regions of Europe. Most European orchids also grow in grasslands and mires, and so we hypothesized that in countries where these habitats are more widespread, the threat level of orchids would be lower. In contrast, countries with a greater extent of anthropogenic land cover should raise threat level.

## Material and Methods

To include a diverse range of economic systems, Europe has, in the context of this paper, been defined as broader than the European Union. However, not all eastern region countries have introduced new red lists. In total, red lists from 27 countries compiled during the last 15 years were quantified and analyzed (Table A1), the majority of which have applied the latest IUCN red list categories. As orchids are one of the best studied plant groups and IUCN categories are based on clear quantitative criteria, we presume the data to be robust enough for further analysis. To achieve taxonomic uniformity throughout this wide range of countries, the World Checklist of Selected Plant Families (WCSP, 2013) was used to identify synonyms. Only the species level was taken into account. If a particular red list separates subspecies, then the threat level was defined as threat of the least threatened subspecies in order to avoid the overestimation of threat. The final list contained 166 species. Data on nectar and life form were obtained from floras.

Threat categories were quantified as follows: 5, regionally extinct – no known individuals remaining in the wild in the country; 4, critically endangered (CR) – extremely high risk of extinction; 3, endangered (EN) – high risk of extinction in the wild; 2, vulnerable (VU) – high risk of endangerment in the wild; 1, near threatened (NT) – likely to become endangered in the near future; 0, least concern (LC) – lowest risk, as it does not qualify for a more at risk category; and data deficient – not enough data to make an assessment of its risk of extinction, but most probably it is threatened. The category LC, in some cases not shown in the national red list, was defined with the help of local floras. Dulvy et al. (2014) in their extinction model of sharks and rays used similarly 0 for LC, but did not differentiate in between endangered categories. According to the IUCN, the main criteria for listing a particular species are population reduction, decline of geographic range, small population size and rapid decline, or very small, restricted population (IUCN, 2001). However, some countries (e.g., Lithuania, Luxembourg, Ukraine) published their red lists within the last 15 years using the older “rare” category (this category is not strictly connected to threat and so is not used any further in the new IUCN system). We counted all incidents of “rare” as 1, or NT.

### Landscape analysis

Based on the framework provided by the European Environmental Stratification described in Metzger et al. (2005), countries were aggregated into four regions. The European Environmental Stratification was built based on 20 relevant climatic and geomorphological variables. The main environmental gradients were extracted with principal components analysis and subsequently clustered with the ISODATA algorithm, resulting in 84 environmental strata, aggregated into 13 environmental zones (EnZs). Prior to performing PCA and ISODATA, the variables were standardized by subtracting the mean of the distribution and dividing this difference by the standard deviation of the distribution. This methodology provides an unbiased division of Europe's environment because it is entirely based on statistical procedures. The European Environmental Stratification therefore sets an appropriate framework for the aggregation of countries into homogeneous groups. The share of each EnZ within every country was calculated, and Grouping Analysis (ArcMap 10.1. ESRI, 380 New York Street, Redlands, CA 92373‐8100, USA) subsequently applied to the countries layer, using the share of each EnZ as input variables. The Grouping Analysis tool performs a classification procedure that finds natural clusters in the data using a *K* means algorithm for grouping. The *K* means algorithm minimizes the differences among the features (countries) in a group when the groups are built. The countries were aggregated into four regions: north, central, Atlantic, south (Table A3).

The area extents of land cover types for each country were calculated based on the CORINE Land Cover 2006 dataset. The CORINE Land Cover dataset is organized into three hierarchical levels; the minimum mapping unit is 25 ha; the minimum width is 100 m and scale 1:100,000 European Environment Agency (EEA), 2007. Because the CORINE Land Cover database offers a homogeneous land cover classification of Europe, we avoided the complex and costly process of aggregating and harmonizing different land cover datasets.

The four land cover categories that most affect orchids (Feldmann and Prat 2011) were defined for the land cover analysis based on the CLC classification: artificial surfaces (level 1, CLC code 1), pastures (level 3, CLC code 231), shrub and/or herbaceous vegetation associations, hereinafter referred to as “grassland” (level 2, CLC code 32), forests (level 2, CLC code 31), and inland wetlands (level 2, CLC code 41). For every country, the total area of each land cover category was calculated in ArcMap 10.1, based on the CORINE Land Cover 2006 seamless vector map. To adequately compare the share of the land cover classes among countries, the extent of each land cover class in each country was calculated as a percentage of the total area of that particular country.

From the original set of 27 countries, Georgia, Greece, Ukraine, Moldova, and Belarus were excluded from the land cover analysis, either because data were missing from complete CLC2006 coverage or because the country had not participated in the CLC project by the time the analysis was undertaken.

### Data analysis

We assessed what factors determined the extinction threat of orchid species as measured by IUCN threat category using ordinal mixed models. Here, we used the *clmm2* function from package *ordinal* (Christensen 2015) in R 3.2.2 (R Core Team, 2015), to fit IUCN threat level as an ordinal, dependent variable in a cumulative link mixed model with orchid subfamily as a random effect. Fixed factors tested include growth form (tuberous vs. rhizomatous), the presence of nectar, geographic region, and the number of countries in which the species was found, as well as four‐two‐way interactions (presence of nectar × region, presence of nectar × number of countries, growth form × region, and growth form × number of countries). This model served as a global model for exhaustive model selection, yielding 31 tested models. The model with the lowest Akaike information criterion (AIC) was deemed the best‐fit model and used for inference, and the strength of fixed effects was determined via likelihood ratio tests of nested models (Table 1). Because this analysis included subfamily as a random factor with only three levels, of which one subfamily contained a single species, we also repeated this analysis using genus instead of subfamily as the random factor. However, this analysis yielded qualitatively the same best‐fit model as with subfamily, and so we do not present those results here.

**Table 1:** Top ten models determining IUCN extinction risk status. Models ordered from the lowest AIC (best‐fit model) and higher. Subfamily was included as a random effect in all models and so is not shown

No.	Model	AIC	AIC weight
1	Tuber + Nectar + Region + No_countries + Tuber × No_countries + Nectar × no_countries	4063.65	0.574
2	Tuber + Nectar + Region + No_countries + Tuber × Region + Tuber × No_countries + Nectar × no_countries	4066.33	0.150
3	Tuber + Nectar + Region + No_countries + Nectar × no_countries	4066.75	0.122
4	Tuber + Nectar + Region + No_countries + Tuber × Region + Nectar × no_countries	4068.01	0.065
5	Tuber + Nectar + Region + No_countries + Nectar × Region + Tuber × No_countries + Nectar × no_countries	4069.54	0.030
6	Tuber + Nectar + Region + No_countries + Tuber × No_countries	4069.65	0.029
7	Tuber + Nectar + Region + No_countries + Tuber × Region + Tuber × no_countries	4072.16	0.008
8	Tuber + Nectar + Region + No_countries + Tuber × Region + Nectar × Region + Tuber × No_countries + Nectar × no_countries	4072.24	0.008
9	Tuber + Nectar + Region + No_countries + Nectar × Region + Nectar × no_countries	4072.59	0.007
10	Tuber + Nectar + Region + No_countries + Tuber × Region + Nectar × Region + Nectar × no_countries	4073.88	0.003

AIC, Akaike information criterion.

We also asked to what extent the land cover of a country influences the IUCN status of its orchid flora. We performed two analyses to answer this question. In the first, we performed a general linear model in which the mean IUCN level of all orchid taxa occurring in a country was a function of region within Europe, total CORINE land cover, total forest land area, total pasture land area, total scrub and grassland area, total inland marsh area, total peat bog area, and total artificial surface area, as well as two‐way interactions between region and forest area, scrub and grassland area, pasture area, and artificial surface area. We used a Gaussian link function in this generalized linear model because mean threat level was approximately normally distributed. In the second analysis, we developed a Poisson‐distributed general linear model of the number of threatened orchid species (i.e., IUCN rank of two or higher) within each country, as a function of the same factors as above. We then used the *dredge* function in the *MuMIn* package (Bartoń 2014) in *R* (R Core Team, 2015) to develop and compare via AIC all nested models within these global models, with the model with the lowest AIC in each case used as the model for inference. We hypothesized that threat level and the number of threatened species would be lower in countries with greater area in grassland and forest, because European orchids are predominantly grassland and forest species. In all cases, we used region as a fixed rather than as a random factor because we included most European countries in our analyses.

## Results

The analysis of threat level of 166 species (Table A2) from 27 European countries revealed that only eight percent of the species were characterized by an average threat level above the endangered category (three), while 18% have an average threat level below the near threat category (one). On the basis of average threat level, among the most threatened species one can find several endemic species including *Cephalanthera caucasica* Kraenzl*., Dactylorhiza kalopissii* E.Nelson and *Gymnadenia carpatica* (Zapal.) Teppner & E.Klein*,* but also species occurring in several or even many countries such as *Liparis loeselii* (L.) Rich.*, Herminium monorchis* (L.) R. Br.*, Neottianthe cucullata* (L.) Schltr.*, Epipactis placentina* Bongiorni & Grünanger*,* and *Spiranthes aestivalis* (Poir.) Rich. Among nonthreatened species, there are those with a really widespread range, such as *Neottia nidus‐avis* (L.) Rich. and *N. ovata* (L.) Bluff & Fingerh. (formerly *Listera*), but also several species with restricted distribution area including *Epipactis rivularis* Kranjcev & Cicmir*, E. turcica* Kreutz, or *Dactylorhiza baumanniana* J.Hölz &Künkele.

**Figure 1:**
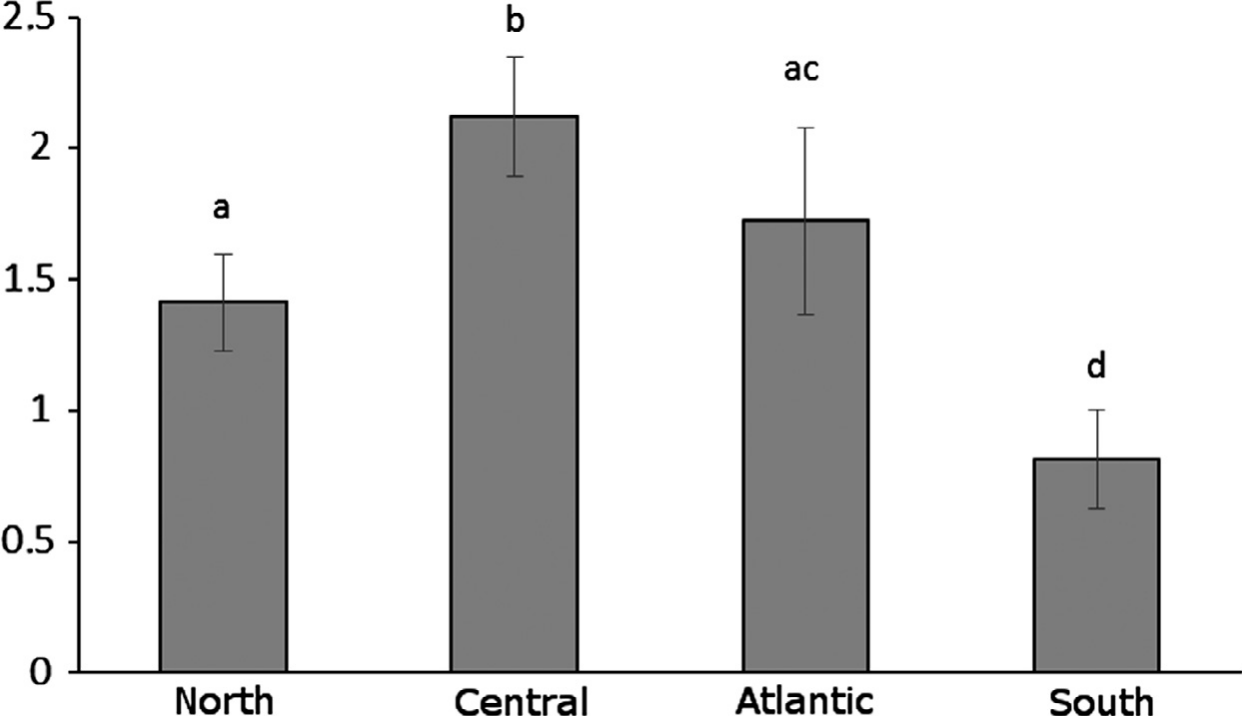
Average threat level (quantified from national and regional red lists: 0 least concern, 1 near threat/rare/data deficient, 2 vulnerable, 3 endangered, 4 critically endangered, 5 regionally extinct) of orchid species in four different regions of Europe (with significant differences calculated with log‐transformed values in the model).

External factors strongly determined the threat level (Table 2). When we divided Europe into four regions, region exerted a significant impact on threat (*χ* ^2^ = 388.81; df = 3; *P *<* *0.0001). Different geographic regions of Europe differed by their orchids’ threat level: The highest was in central Europe, followed by the Atlantic, followed by northern Europe. The lowest threat level was in southern Europe (Fig. 1). The number of countries an orchid was found also exerted a significant impact (*χ* ^2^ = 52.08; df = 3; *P *<* *0.0001), with increasing number of countries contributing to lower risk level.

**Table 2:** Parameter estimates from the best‐fit ordinal mixed model of IUCN risk status. Status as tuberless, nectarless, and with a distribution in northern Europe served as baselines with 0 estimates, and so are not included in the table

Parameter (fixed effect)	Estimate	SE	*z*‐Value	*P* ≤
Presence of tuber	−0.078	0.278	−0.281	0.778
Presence of nectar	0.285	0.255	1.118	0.263
Number of countries	−0.043	0.014	−3.047	0.002
Region: central Europe	0.830	0.150	5.540	0.0001
Region: Atlantic Europe	0.324	−0.21	1.543	−0.123
Region: southern Europe	−1.069	0.172	−6.230	0.0001
Presence of tuber × the number of countries	0.031	0.014	2.263	0.024
Presence of nectar × the number of countries	0.038	0.013	−2.828	0.005

Random effect	Variance			
Subfamily	0.281			

Orchid biology exerted a number of important influences on threat level, as well. Nectarless species were significantly less threatened than nectariferous (*χ* ^2^ = 28.15; df = 2; *P *<* *0.0001), while rhizomatous species were significantly more threatened than tuberous (*χ* ^2^ = 7.99; df = 1; *P *=* *0.025). However, significant interactions between nectar and the number of countries (*χ* ^2^ = 7.40; df = 1; *P *=* *0.005), and between tuber and the number of countries (*χ* ^2^ = 5.10; df = 1; *P *=* *0.024), suggest that these patterns reverse in taxa found in three or more countries for rhizomatous vs. tuberiferous orchids and in taxa found in eight or more countries in nectariferous vs. nectarless orchids. Taxonomically, the subfamily Cypripedioideae with its single representative was more threatened on average than the Epidendroideae and Orchidoideae (*P *=* *0.007; *P *=* *0.0006), respectively, while there was no statistical difference between the latter two (*P *=* *0.09).

Mean IUCN threat level per country varied by regions and by the amounts of land area in scrub and grassland. Particularly, threat level decreased with increasing scrub and grassland area (*P *=* *0.0411) and was highest in region 2, followed by region 3, region 1, and region 4. The number of threatened orchid species per country also varied regionally and in the same order as the mean IUCN threat level. The number of threatened orchid species per country increased with scrub and grassland area (*P *<* *0.0001), lending special importance to that factor, but also increased with artificial surface area (*P *<* *0.0001), and decreased with inland marsh area (*P *<* *0.0001).

## Discussion

Following half a century of development, the red list has become not only a key tool in conservation (Rodrigues et al. 2006), but has also enabled scientific analysis of status and trends in several groups of organisms on a large scale (Brummitt et al. 2015). Besides mammals, birds, and amphibians, orchids are certainly among the most assessed vascular plants due to the vast numbers of amateurs taking an interest in the collection of data on their distribution. The current IUCN red list categories are based wholly on the threat level of species as evaluated by a set of quantitative criteria. These categories have been applied in many countries in the process of creating their national red lists, which provide fundamental information on trends in biodiversity loss (Zamin et al. 2010).

The threat level for European orchid species is high, reaching on average the level of vulnerable (IUCN category), although only eight percent are on average, highly endangered. Although it is to be expected that stenoendemic species are rare and vulnerable, not all endemic species are necessarily endangered or even threatened (Kruckeberg and Rabinowitz 1985). Indeed, some narrowly endemic plants may be locally very persistent and common (Vogt‐Schilb et al. 2013). This could explain why endemic species occur at both ends of our list.

Among the most threatened species, we detected a few that occurred across most European countries. Still, on average, the more countries a species inhabits, the less threatened it is. Therefore, the fate of such species as *L. loeselii* and *H. monorchis*, which occur in most countries analyzed but are endangered in almost all of them, should be very seriously treated. We note particularly that the processes determining rarity at different scales may often differ (Rabinowitz et al., 1986), as regional distributions relate more to dispersal and colonization patterns than local distributions, which are likely governed more by population ecological factors. Furthermore, while the global distribution area of *H. monorchis* is largely within the region considered in our study (Hultén and Fries 1986), it is also declining in the neighboring country of Russia (Efimov 2011). While *L. loeselii* is at least protected in the EU as a species of community interest, *H. monorchis* is covered only through national attempts at conservation.

Our analysis of threat level in the three orchid subfamilies represented in our list showed that Cypripedioideae is significantly more threatened than the other two subfamilies, whose difference was not significant. However, we know that Cypripedioideae in Europe contains only one species *Cypripedium calceolus* L., and it is quite genuinely threatened. Thus, sampling restrictions prevent us from considering evolutionary history at the subfamily level to be important here. Furthermore, the analysis at genus level did not show any differences either.

Of particular concern in the conservation of orchids are their symbioses with pollinators and fungi (Tremblay et al. 2005; Waterman and Bidartondo 2008; Swartz and Dixon 2009). Orchids are often nectarless and sometimes depend on rather specific pollinators (Cozzolino and Widmer 2005). In addition, they are often rather specialized on particular mycorrhizal fungi, without which they cannot germinate (Rasmussen 1995; Shefferson et al. 2007). Such complicated reproduction biology may become problematic especially under changing environmental conditions resulting, for example, from modification to management regimes or climate change (Robbirt et al. 2011, 2014; Molnár et al. 2012). Specific biological traits may increase the threat level of species (Pövry et al. 2009). The results of our study show that the type of pollination syndrome – the presence of nectar – has a significant effect, although it is different in widely distributed and more local species.

Nectarless deceivers have a much lower fruit set than nectariferous rewarding species (Neiland and Wilcock 1998). However, several attempts to find a greater decline of nectarless species across a smaller geographic range (Jacquemyn et al. 2005; Kull and Hutchings 2006) have been unsuccessful, whereas the rarity of orchids in the British Isles has been shown to be connected to the availability of nectar in these species (Neiland and Wilcock 1998). In response to climate change, the flowering time of nectarless orchids has shifted earlier (Molnár et al. 2012), more so than that of nectariferous species, and the match with the phenology of pollinators may have become broken, such as in the case of the sexually deceptive *Ophrys sphegodes* Mill. (Robbirt et al. 2014). Furthermore, fragmentation has increased the isolation of populations, with deceiving species even more sensitive to decreased gene flow than nectariferous species (Gijbels et al. 2015). Intriguingly, although these patterns are now clear with regard to pollination, the link between mycorrhizal specialization and rarity is far less clear, with some of the most strongly specialized orchids often being the least rare (Shefferson et al. 2005; Phillips et al. 2011).

The rather severe pollination biology of many orchids may suggest that clonal reproduction would be linked to a lower threat level, but in a whole flora analysis of persistence of species in the UK and Estonia, clonality did not give any advantage (Laanisto et al. 2015). However, in a comparison of orchids in the same countries, tuberous species were more threatened than rhizomatous species (Kull and Hutchings 2006). The rhizomatous species tend to be more tolerant of unfavorable conditions and importantly, as a result, are long‐lived (Van Groenendael et al. 1997). They do not need to go through the complicated phases of sexual reproduction so frequently. Probably the loss of pollinators and good germination sites may be hidden in this factor.

Due to the rather more unfavorable climatic conditions in northern Europe, we had expected to see that orchids existing at the edge of their distribution area would be more threatened than in other European regions. While it was true that orchids were the least threatened in the Mediterranean Europe, the threat level in central Europe was significantly higher than in the northern Europe or in any other European region. In highly industrial central Europe, the area under artificial land cover is huger than in the north or south, but not higher than in the Atlantic region. The percentage of the area of grasslands and pastures, which provide important habitats for many orchid species, had a significant impact on the threat level. It is possible that the very high share of pastures in the Atlantic region compensates for the large share of the artificial surfaces. In central Europe, orchids suffer as a result of high urbanization levels and very intensive agriculture. The CORINE is the only dataset providing a comprehensive overview of land cover at the European level, enabling comparisons such as that described in the present article (Buttner 2014). However, CLC categories are not pure at fine scale (Gallego and Bamps 2008), and it tends to be too focused on agriculture instead of seminatural environments. The sheer scale of the CORINE project results in unavoidable generalization in the definition of classes (Bach et al. 2006). Such is the case for the land cover type “inland wetlands,” a broad classification that contains a very wide range of marshes and bogs, even peat extraction areas (Buttner 2014). Because orchids are mainly associated with calcareous soils, the results from the proportion of inland wetlands and orchids threat‐level analysis should be interpreted with caution.

This study demonstrated that the application of the current IUCN categories and criteria to national red lists enables the results to also be applied across several types of broader threat analyses of different groups of organisms and regions. We believe that further incorporation of these red list data with more specific ecological knowledge, such as mycorrhizal associations, can lead to a more accurate classification of threat in rare and endangered species worldwide, and not just for orchids. We also believe that our results should spur greater efforts to conserve the orchids particularly of central Europe and of European wetlands and their native orchid species.

## Conflict of Interest

None declared.

## Figures and Tables

**Table A2:**
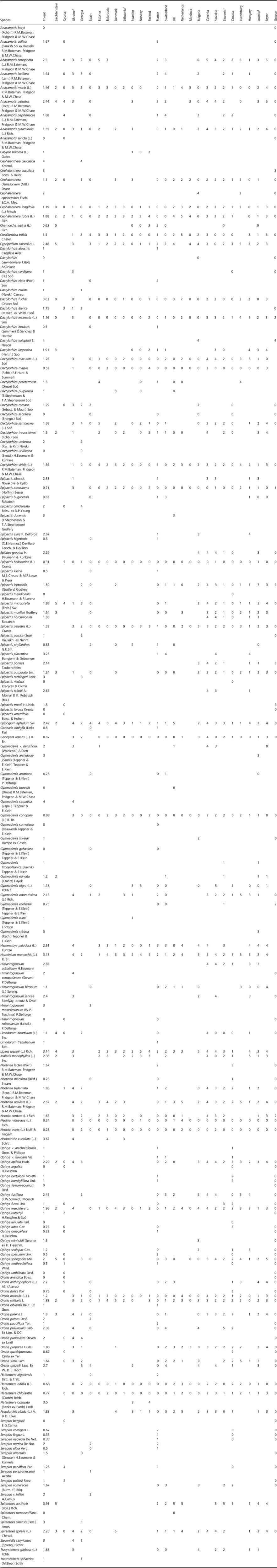
Quantified average threat level of European orchid species on the basis of 27 national and regional red lists (^1^older or modified IUCN categories).

## Appendix

National red lists used in the analysis (Table A1), average threat levels of species (Table A2), and countries aggregated into four regions (Table A3).

**Table A1:** National and regional red lists used in the analysis

Austria – Niklfeld, H., L. Schratt‐Ehrendorfer. 1999. Rote Liste gefährdeter Farn‐ und Blütenpflanzen (Pteridophyta und Spermatophyta) Österreichs
Belarus – Кpacнaя книгa Pecпyблики Бeлapycь. 2006. Mиниcтepcтвo пpиpoдныx pecypcoв и oxpaны oкpyжaющeй cpeды Pecпyблики Бeлapycь. Published on the Internet; http://redbook.minpriroda.by/about.html (11 January 2013)
Bulgaria – Petrova, A., V. Vladimirov (Eds.). 2009. Red List of Bulgarian Vascular Plants. Phytologia Balcanica 15:63–94
Croatia – Flora Croatica Database. 2004. University of Zagreb. Published on the Internet; http://hirc.botanic.hr/fcd/search.aspx (01 March 2013)
Cyprus – Tsintides, T. C.S. Christodoulou, P. Delipetrou, K. Georghiou, (Eds.). 2007. To kokkino bib*λ*io th*σ* x*λω*pi*δ*a*σ* th*σ* Ky*π*poy. The Red Data Book of the Flora of Cyprus. Lefkosia
Czech Republic – Holub, J., F. Procházka. 2000. Red list of the flora of the Czech Republic (state in the year 2000). Preslia, Praha, 72
Denmark – Wind, P., S. Pihl, (Eds.). 2004. Den danske rødliste. Danmarks Miljøundersøgelser, Aarhus Universitet. Published on the Internet; http://redlist.dmu.dk (11 January 2013)
Estonia – eElurikkus. 2008., Eesti ohustatud liikide punane nimestik. Published on the Internet; http://elurikkus.ut.ee/prmt.php?lang=est (11 January 2013)
Finland – Rassi, P., E. Hyvärinen, A. Juslén, I. Mannerkoski, (Eds.). 2010. Suomen lajien uhanalaisuus ‐ Punainen kirja 2010. The 2010 Red List of Finnish Species. Edita Prima Oy, Helsinki
France – UICN France, MNHN, FCBN, SFO. 2010. La Liste rouge des espèces menacées en France ‐ Chapitre Orchidées de France métropolitaine, Paris
Georgia – Red list of Georgia. 2006. Published on the Internet; http://moe.gov.ge/index.php?lang_id=ENG&sec_id=48&album_id=3&info_id=#seegal (01 March 2013)
Germany – Bavaria ‐ Rote Liste der gefährdeten Tiere und Gefäßpflanzen Bayerns. Bayerisches Staatsministerium für Umwelt, Gesundheit und Verbraucherschutz, 2005. Published on the Internet; http://www.lfu.bayern.de/natur/rote_liste_pflanzen/index.htm
Greece – Dimopoulos, P., Th. Raus, E. Bergmeier, Th. Constantinidis, G. Iatrou, S. Kokkini, A. Strid, D. Tzanoudakis. 2013. Vascular plants of Greece: an annotated checklist. Botanischer Garten und Botanisches Museum, Berlin‐Dahlem, Freie Universität Berlin; Athens: Hellenic Botanical Society. Englera 31, 1–370
Hungary – Kiraly, G., (Ed.). 2007. Vörös Lista. A magyarországi edényes flóra veszélyeztetett fajai. Red list of the vascular flora of Hungary. Löver Print, Sopron
Liechtenstein – Broggi, M.F., E. Waldburger, R. Staub. 2006. Rote Liste der gefährdeten und seltenen Gefässpflanzen des Fürstentums., Amtlicher Lehrmittelverlag, Vaduz
Lithuania – Rašomavičius, V., (Ed.). 2007. Red Data Book of Lithuania. Lutute Publishing, Vilnius
Luxembourg – Colling, G. 2005. Red List of the Vascular Plants of Luxembourg. Published on the Internet; http://ps.mnhn.lu/recherche/redbook/vascplants/default.htm (11 January 2013)
Moldova – Sârbu, I., A. Oprea, I. Lupu. 2005. Specii de plante vasculare ameninţate din Moldova, Asociaţia Dendro‐Ornam. Anastasie Fătu, Iaşi: 6–98
Norway – Kålås, J.A., Å. Viken, T. Bakken. 2006. Norsk Rødliste 2006. 2006 Norwegian Red List. Artsdatabanken, Trondheim
Slovakia – Baláž, D., K. Marhold, P. Urban, (Eds). (2001) Červený zoznam rastlín a živočíchov Slovenska. Ohrana Prírody 20 (Suppl):48–81
Slovenia – Anonymous. 2002. Pravilnik o uvrstitvi ogroženih rastlinskih in živalskih vrst v rdeči seznam. Uradni list RS 82/2002. Uradni list, Ljubljana
Spain – Bañares, Á., G. Blanca, J. Güemes, J.C. Moreno, S. Ortiz, (Eds.). 2010. Atlas y Libro Rojo de la Flora Vascular Amenazada de España. Adenda 2010. Dirección General de Medio Natural y Política Forestal (Ministerio de Medio Ambiente, y Medio Rural y Marino)‐ Sociedad Española de Biología de la Conservación de Plantas, Madrid
Sweden – Gärdenfors, U., (Ed.). 2010. Rödlistade arter i Sverige 2010. ArtDatabanken SLU, Uppsala
Switzerland – Moser, D.M., A. Gygax, B. Bäumler, N. Wyler, R. Palese. 2002. Rote Liste der gefährdeten Arten der Schweiz. Farn‐ und Blütenpflanzen. Bundesamt für Umwelt, Wald und Landschaft, Bern
The Netherlands – Besluit van de Minister van Landbouw, Natuur en Voedselkwaliteit van 28 augustus 2009, 25344, houdende vaststelling van geactualiseerde Rode lijsten flora en fauna. Published on the Internet; http://www.rijksoverheid.nl/documenten-en-publicaties/besluiten/2009/09/07/besluit-vaststelling-geactualiseerde-rode-lijsten-flora-en-fauna.html (15 February 2013)
Ukraine – Didukh, Ya.P., (Ed.). 2009. Chervona knyga Ukrainy. Roslynnyi svit. Globalkonsaltyng, Kyiv
United Kingdom – Cheffings, C.M., L. Farrell, (Eds.). 2005. The Vascular Plant Red Data List for Great Britain. Species Status 7: 1–116. Joint Nature Conservation Committee, Peterborough

**Table A3:** European countries aggregated into four regions

Region	Countries^a^
North	Norway, Sweden, Finland, Estonia, Lithuania, Belarus
Central	Switzerland, Bulgaria, Moldova, Czech Republic, Slovakia, Luxembourg, Hungary, Ukraine, Austria, Liechtenstein, Germany (Bavaria)
Atlantic	United Kingdom, Netherlands, Denmark
South	Spain, France, Cyprus, Slovenia, Croatia, Greece

a As Georgia was not covered by the stratification, it was excluded from this analysis.
